# Innovative Ante-Natal Care: Healthy Teeth Healthy Baby, An Interprofessional Collaboration Initiative In Indonesia

**DOI:** 10.1093/eurpub/ckac129.260

**Published:** 2022-10-25

**Authors:** I Adyatmaka, H Lee

**Affiliations:** 1 Department of Dentistry, Christian Maranatha University, Bandung, India; 2 Oral Health Workgroup, WFPHA, Geneva, Switzerland

## Abstract

**Background and problem:**

SDG 3.1 sets out that by 2030, the global maternal mortality ratio (MMR) should be reduced to 70 per 100,000 lives and no country should have MMR above 140 per 100,000 lives. Indonesia still has an MMR of 177 by 2017 data by the World bank. Urgent action is needed to improve the health and survival of women and babies. The main causes of MMR in Indonesia are Hemorrhage, followed by pre-eclampsia, and others, related with diabetes, stroke, etc.

**Importance:**

Previous studies have shown the association between oral dysbiosis and pre-eclampsia, preterm birth and low birth weight. Oral microbiome of the mother being a key player in pregnancy outcomes. Periodontal disease is considered a possible risk factor for the health of the mother and the newborn.

**Solution:**

Program goal of this program, “Innovative AnteNatal Care, Healthy Teeth, Healthy Baby”, is to create the ecology right from the start, by incorporating oral health diagnosis and prevention into routine antenatal care. This approach achieves a healthy oral condition that can improve systemic health during pregnancy. The program also achieves a healthy and resilient family through oral health empowerment and delivering healthy babies (superior generation 2045- President Joko Widodo). Processes included avocation to provincial health districts, initial survey of the perception and oral health status of pregnant mothers, module preparation, and socialization to health districts and related officers. The program has trained midwives and dental providers and secured governmental budgets for sustainable activities, including socialization and assistance to pregnant mothers and their husbands, field penetration by health professionals, and evaluation of the programs.

